# Atypical origin, structure and arrangement of secondary tracheary elements in the stem of the monocotyledonous dragon tree, *Dracaena draco*

**DOI:** 10.1007/s00425-016-2593-4

**Published:** 2016-09-01

**Authors:** Joanna Jura-Morawiec

**Affiliations:** Polish Academy of Sciences Botanical Garden, Centre for Biological Diversity Conservation in Powsin, Prawdziwka 2, 02-973 Warsaw, Poland

**Keywords:** Intrusive growth, Monocotyledons, Secondary growth, Tracheids, Vascular bundles

## Abstract

**Tracheary elements within the secondary body of a dragon tree shared features characteristic of fibres. Their considerable intrusive growth resulted in a rigid network with a braid-like arrangement which contributes towards the tree-like form of the plant.**

Monocot cambium gives rise to xylem and phloem which become organized into vascular bundles. The xylem consists entirely of tracheids, and these undergo considerable intrusive elongation during their development, unlike the tracheids of conifers and those of vesselless dicotyledons. Monocot tracheids have not been fully investigated, and our understanding of their structure is incomplete. Therefore, in this study the degree of variation in the structure and arrangement of secondary tracheary elements of monocots were determined, based on the *Dracaena draco* stem. In addition, its mechanical and physiological implications were discussed. Analysis of series of thin serial sections and macerations of the immature and fully developed tracheids showed that the course of intrusive elongation of tracheids was determined by the spatial relationship that exists between the growing tracheid and surrounding cells, and was not usually parallel to the stem axis. It influenced the shape of tracheids, as well the cross-sectional shape of vascular bundles. Tracheids become twisted or even interwoven and so, their ends do not join with the ends of other tracheids. The complexity of the tracheid network, that functions both in transport and mechanical support, seems to have a major impact on the tree-like growth habit of *D. draco*.

## Introduction

Secondary growth of monocotyledonous plants is related to the activity of the lateral meristem, referred to as the monocot cambium (Carlquist 2012). This activity gives rise to the phloem and xylem of the secondary vascular bundles, as well as to the ground parenchyma in which they are embedded, and these tissues, collectively, constitute the so-called monocot wood (Tomlinson and Zimmermann 1967). Addition of secondary bundles to stems by the monocot cambium is an adaptation which has enabled monocotyledonous plants to achieve great tree-like forms, exceeding some 20 m in height (as is the case for *Dracaena draco*), and these are capable of living as much as 700 years (Byström 1960; Symon 1974). The key to the evolutionary success of these plants is also the presence of imperforate tracheary elements, i.e. the tracheids in secondary bundles, since these cells have a dual function, namely, the transport of water to the leaf canopy and the ability to support it physically. These tracheids are very long, often at least 38- or 40-fold longer than the mother cells from which they are derived (Scott and Brebner 1893; Cheadle 1937). Such tracheid length is achieved by intrusive growth (Waterhouse 1987). It is known that elongation by intrusive growth is not a typical feature of tracheids. The tracheids of conifers and those of vesselless dicotyledons usually resemble cambial initials in both length and width, and their extra-cambial elongation is estimated to be approx. 5 % (Bailey and Tupper 1918; Bailey 1944, 1957; Larson 1994). The intrusive growth of monocot tracheids exceeds even that of dicot xylem fibres, with extra-cambial intrusive elongation having been reported to be some ten-fold longer than the length of cambial initials (Larson 1994; Evert 2006). The range of tracheid elongation growth in monocots is comparable to that of the secondary phloem fibres of hemp, which, on average are 30-fold longer than the cambial initials (Snegireva et al. 2010, 2015). They are relatively wide (Carlquist 2012), vary in shape, and have oblique, narrow-aperture pits with distribution that resemble those usually found in fibres (Waterhouse 1987). The mechanical properties of monocot wood are comparable to those of dicot wood of similar density, as demonstrated for *D. mannii* (Torelli and Trajković 2003), the tracheids contributing significantly towards this mechanical strength.

Tracheary elements of monocotyledons with secondary growth have not yet been fully investigated and our understanding of their structure is incomplete. Therefore, the aim of this study was to gain more insight into the formation, structure and arrangement of tracheids originating from the monocot cambium of *Dracaena draco* stem. For this purpose, an anatomical analysis, involving serial sectioning and macerations of immature and fully developed tracheids was conducted. The study provides some new information regarding their growth and consequent longitudinal and transverse arrangement within amphivasal vascular bundles. In addition, the mechanical and physiological implications of these results are discussed. This work forms part of an ongoing series of anatomical studies into monocotyledons that display secondary growth (Jura-Morawiec and Wiland-Szymańska 2014; Jura-Morawiec 2015; Jura-Morawiec and Tulik 2015; Jura-Morawiec et al. 2015).

## Materials and methods

### Plant material and its preparation

The data presented in this paper are derived from the stem of a single *D. draco* plant grown under glass at the Polish Academy of Sciences Botanical Garden—CBDC in Powsin. Nevertheless, observed trends relating to the structure and arrangement of tracheids were confirmed by repeating anatomical observations for stems of two further *D. draco* plants grown at Jardín Botánico Canario “Viera y Clavijo” on Gran Canaria. The tissue samples (ca. 2 cm long, ca. 1 cm wide, ca. 1 cm thick) for each were comparable, containing both immature vascular bundles adjacent to the monocot cambium, but with zonation barely visible (Jura-Morawiec 2015), together with mature amphivasal vascular bundles. The samples were fixed in a mixture of glycerol and ethanol (1:1; v/v), then cut into smaller pieces (ca. 3 mm long, ca. 2 mm wide, ca. 2 mm thick), processed for Epon embedding using the method described by Meek (1976) and subsequently cut both tangentially and transversely to form a continuous series of thin (3 µm) sections using a Tesla 490A microtome. The resultant sections were stained with PAS and toluidine blue, and mounted in Euparal. Macerations of xylem elements were prepared according to Franklin (1945) and stained with 0.01 % safranin 0 solution. Prior to this, however, mature xylem, as well as parts of the xylem that had not developed fully, were separated with the aid of an Opta-Tech X2000 stereoscopic microscope and macerated independently. The sections and macerations were examined under transmitted light using an Olympus BX 41 microscope.

### Microscopical analysis

The length of the mother cells of vascular bundles and pit diameter were measured for tangential sections. Tracheid lengths were measured using maceration preparations. For the calculation of means + standard errors using Microsoft Excel, 50 measurements were taken in each case, using a calibrated eye-piece micrometer. The arrangement of tracheids in developing and mature amphivasal bundles was traced using transverse sections for approx. 192 and 312 µm, respectively, along the longitudinal axis of the stem.

## Results

**Fig. 1:**
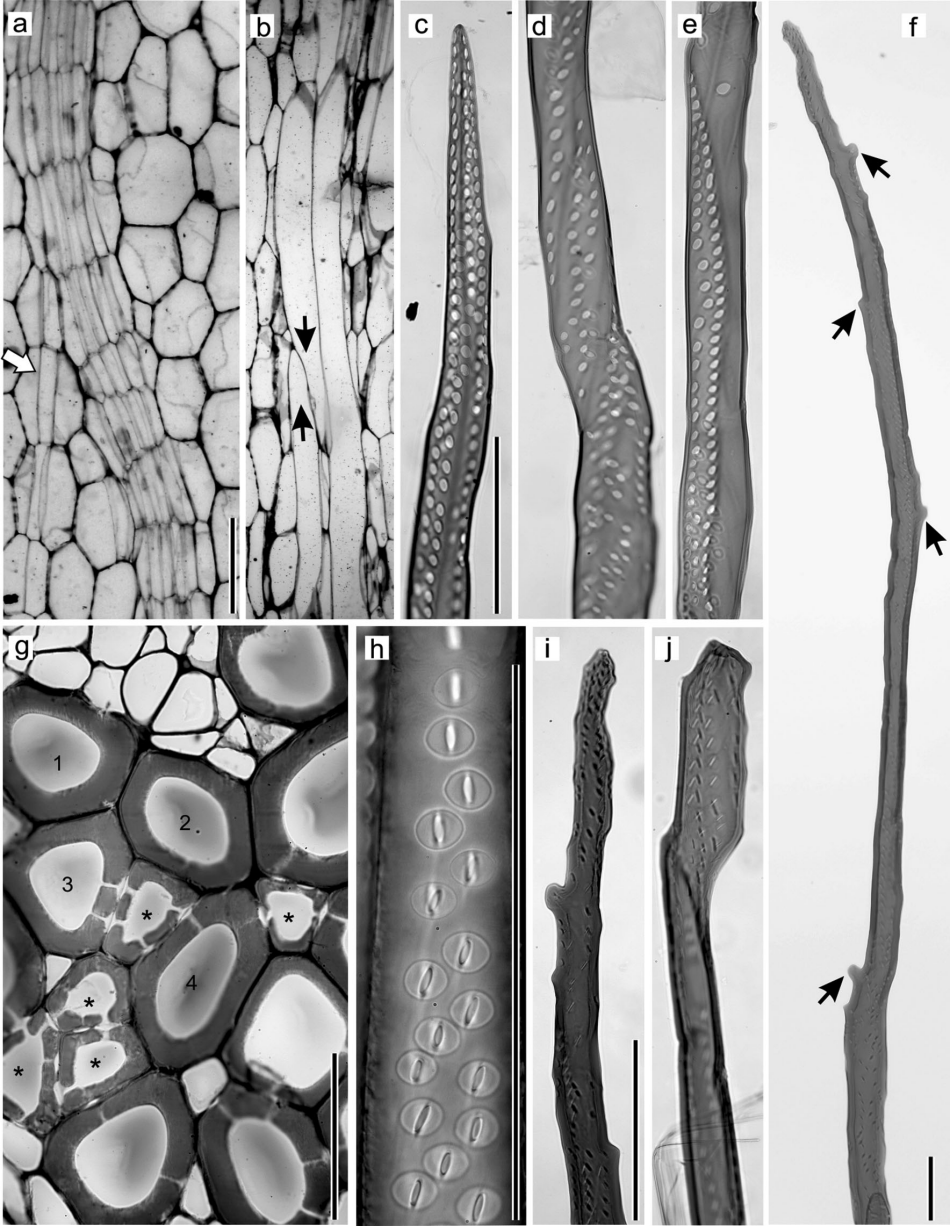
Characteristic features of tracheids of *D. draco* stem. Tangential longitudinal section through zone of mother cells of amphivasal bundles; uniting/separation of vascular bundles indicated by *arrow* (**a**). Early stages of tracheid development in tangential view, tapered tracheid ends marked by *arrows* indicate elongation by intrusive growth (**b**). Parts of macerated, immature tracheids (**c**–**e**). End of macerated, mature tracheid showing protrusions (indicated by *arrows*) (**f**). Transverse section, with tracheid ends marked by *asterisks*. Note the distribution of pits in walls of tracheids numbered 1–4 (**g**). Bordered pits in overlapping contact areas of tracheids (**h**). Ends of mature tracheids showing characteristic shape and pits (**i**–**j**). *Scale bar* 100 µm

In *D. draco* stem, the average length of vascular bundle mother cells (Fig. 1a) was 0.086 ± 0.022 mm. Within each bundle, tracheids were the only elongated elements, and at functional maturity measured, on average, 4.95 ± 0.88 mm in length. Thus, the growth of a tracheid mother cell led to a ~57-fold increase in length. Initiation of intrusive elongation could be recognized by the presence of characteristic tapered ends during the early stages of tracheid development (Fig. 1b). Tracheids that had completed elongation possessed variously shaped ends that were not only tapered, but also displayed characteristic protrusions visible along the entire length of the cell (Fig. 1c, f, i, j). Tracheids possessed pitted walls. Pit distribution was easier to observe in macerated, immature tracheids, when neither secondary wall nor pits were fully developed (Fig. 1c–e). Unlike typical tracheids, those of *D. draco* did not overlap at their ends. Instead, the end wall of one tracheid usually overlapped the body of an adjacent tracheid, and thus, their characteristic pitted contact surfaces could be clearly seen (Fig. 1e, g). Mature tracheids had bordered pits lacking a torus-margo structure (Fig. 1g-j). Pits were circular, about 8 µm in diameter, with elliptic apertures like those shown in Fig. 1h–j. In transverse section, the tracheids were polygonal and compactly arranged within amphivasal bundles (Fig. 1g).

**Fig. 2:**
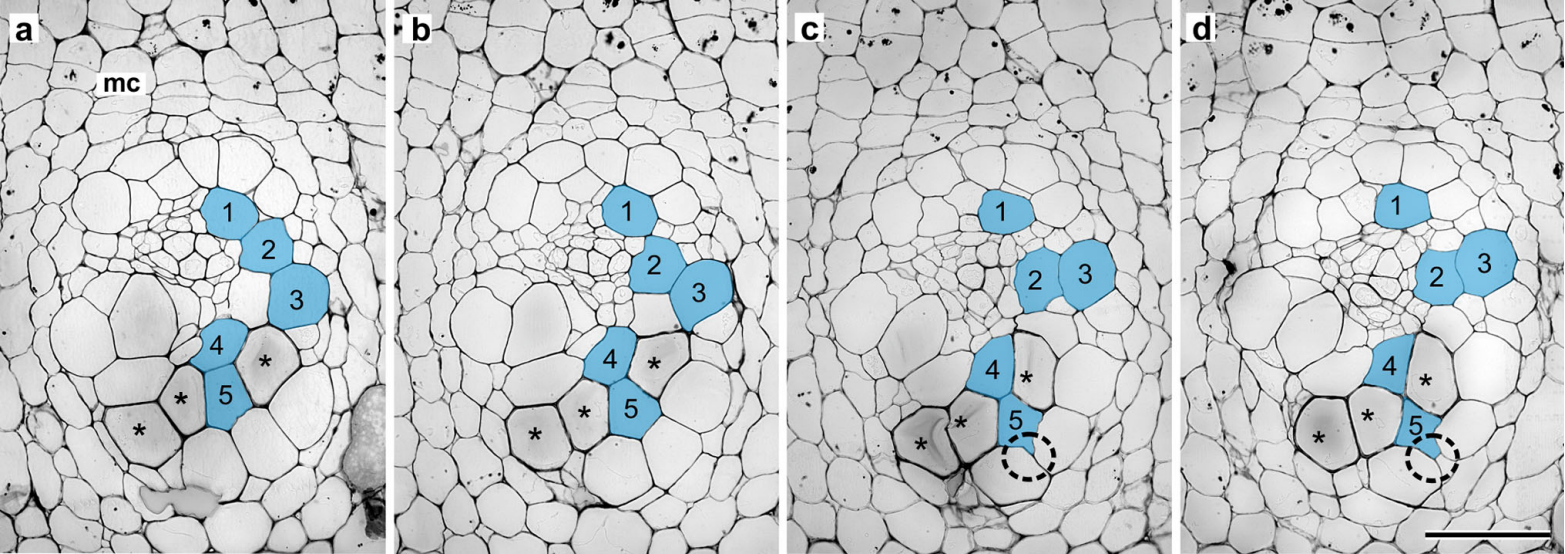
Tracheid growth during amphivasal bundle development. Selected transverse sections from a series of 64 serial sections covering a distance of 192 µm (**a**–**d**). Tracheids that had considerably changed shape/contacts with other cells during the growth phase are numbered (1–5) and *marked blue*. Tracheids displaying first signs of secondary cell wall deposition are marked with *asterisks*. Intrusion by tracheid end is encircled; *mc* monocot cambium. Distances between these selected sections are: 45 µm between **a** and **b**, 81 µm between **b** and **c**, 66 µm between **c** and **d**. *Scale bar* 100 µm

Serial transverse sections of developing amphivasal bundles (i.e. when most tracheids in an analysed bundle have completed the growth stage, and some have begun to show signs of secondary cell wall deposition), revealed that the course of intrusive growth by tracheids is determined by the spatial relationships that exists between the growing tracheid and surrounding cells (Fig. 2). Tracheids are able to elongate in different cellular environments that determine their shape and course of elongation i.e., they may lie adjacent to vascular parenchyma, sieve tube elements, ground (conjunctive) parenchyma or other elongating tracheids. As seen in the example of 1–5 selected tracheids, at planes a–b, tracheid no. 1 abuts tracheid no. 2, whereas at planes c–d, these tracheids are no longer associated with each other and become separated by vascular parenchyma cells. Tracheid no. 3 considerably changes its position relative to tracheid no. 4. In turn, the end of tracheid no. 5 intrudes between the walls of neighbouring tracheids and makes new contact with a cell of ground parenchyma (Fig. 2c, d). Thus, the course that tracheids take does not always run parallel to the longitudinal axis of the stem, but tracheids may become strongly displaced or even twisted relative to each other, as was also shown by macerations (Fig. 1d).

**Fig. 3:**
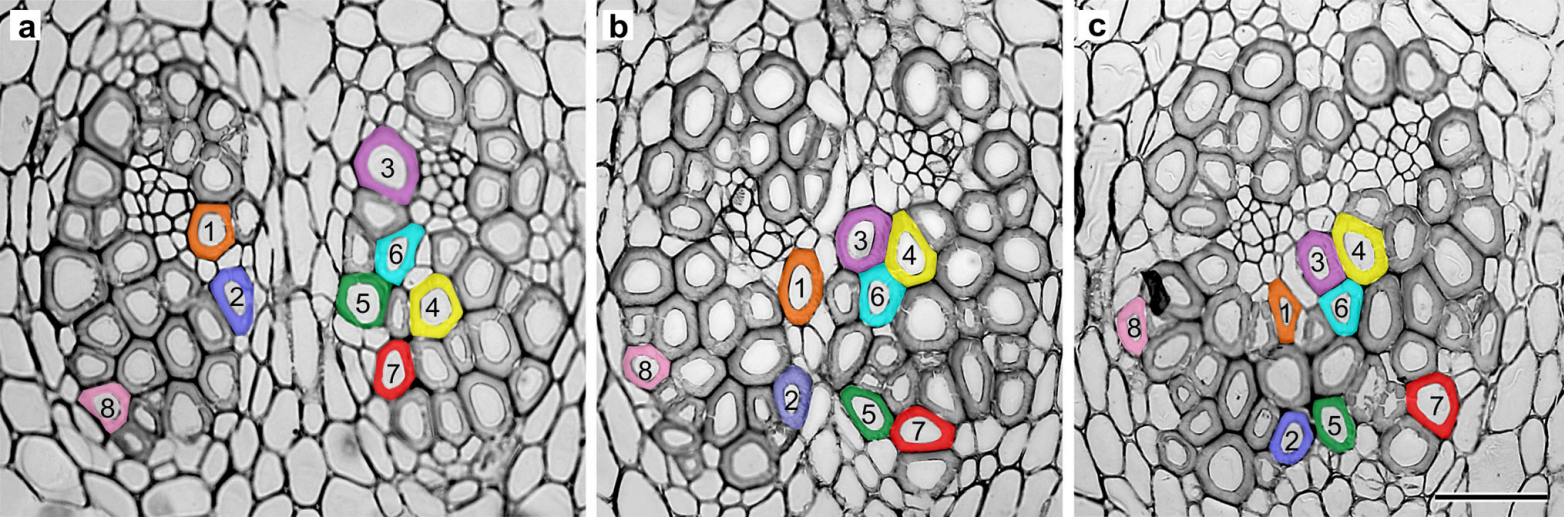
Mature amphivasal vascular bundles that have united tangentially during development. Selected successive transverse sections from a series of 104 sections covering a distance of 312 µm along the stem axis (**a**–**c**). Note changes in position and shape of numbered and coloured tracheids. Distances between these selected sections are: 174 µm between **a** and **b**, 138 µm between **b** and **c**. *Scale bar* 100 µm

Vascular bundles, during their development, may undergo a process of uniting along the length of the stem axis (Fig. 1a). As a result, the number of tracheids within a given vascular bundle, as seen in transverse section, increases significantly from 33 ± 5, to as many as ~50–70. The ends of tracheids grow in opposite directions as they compete for space and this results in considerable change to their shape and arrangement within a given bundle. This has been recorded for mature amphivasal bundles whose courses have been traced along the longitudinal stem axis (Fig. 3). The number of tracheid ends and bodies visible in transverse section at a given plane changes as one passes along the stem axis, since the tracheids do not form a regular column.

## Discussion

### Tracheid growth and its contribution to the structure of vascular bundles

In monocots with dracaenoid type of growth, the tracheids form part of the secondary plant body and their great length is achieved by intrusive growth (Waterhouse 1987). The key features of apical intrusive growth include (a) the occurrence of denser cytoplasm at the ends of elongating cells (Larson 1994), as well as (b) the shape of the cell during early stages of differentiation, i.e. the presence of so-called ‘knees’ (Snegireva et al. 2010). As far back as 1893, Scott and Brebner reported the presence of denser cytoplasm within the pointed ends of *Yucca* tracheids, although these authors considered this to be a symptom of sliding growth. The presence of ‘knees’ during early stages of growth was observed in the present study of *D. draco*. Some tracheids grew also laterally, thus forming characteristic protrusions that are irregularly distributed along the length of the cell. The presence of such protrusions has been recorded for secondary wood fibres of the dicotyledonous tree *Lonchocarpus sericeus.* Their occurrence here, however, was regular and associated with the double-storied arrangement of the vascular cambium, the rays being shorter than the fusiform initials (Jura-Morawiec et al. 2008). Wenham and Cusick (1975) pointed out that cells growing intrusively do so along an intrusive pathway or the route of least resistance. During bundle formation, the ends of some tracheids grow upward, others downward, and thus, the formation of protrusions may be due to contact being made between the ends of two elongating tracheids as they compete with each other for space to grow. Conversely, intrusive elongation of tracheids is also associated with the differentiation of other types of non-elongating cells that lack uniform shape and constitute part of the vascular bundle. This process results in the local formation of intercellular spaces which can be occupied by the growing tips of tracheids, thereby forming protrusions.

The number of tracheids within an amphivasal vascular bundle is dependent on the patterning determined by the meristem from which they are derived and the subsequent intrusive growth of individual tracheids. In *Yucca*, which has collateral secondary bundles, on average, only one cell from each desmogen strand elongates to become a tracheid (Scott and Brebner 1893). These tracheids, by intrusive elongation, join other tracheids formed along the longitudinal axis of the organ, and thereby significantly increase the number of cells in the vascular bundle. This was represented schematically by Waterhouse (1987). Recently, a similar method of bundle formation has been described for bundles of fibres in the secondary phloem of hemp (Snegireva et al. 2015). In turn, a considerable increase in the number of tracheids in an amphivasal bundle of *D. draco* stem indicates the uniting of vascular bundles during development. The vascular bundles are able to unite both tangentially and radially (Scott and Brebner 1893; Zimmermann and Tomlinson 1970). Finally, the number of cells, as well as their arrangement, in a mature vascular bundle, reflect a morphogen gradient governing the pattern of tissue development. Research data into factors that control tracheid length in monocots are lacking. In the case of typical tracheids and fibres intrusive growth is promoted by gibberellin in the presence of auxin (Kalev and Aloni 1988; Aloni 2007, 2015).

### Mechanical and physiological implications

**Fig. 4:**
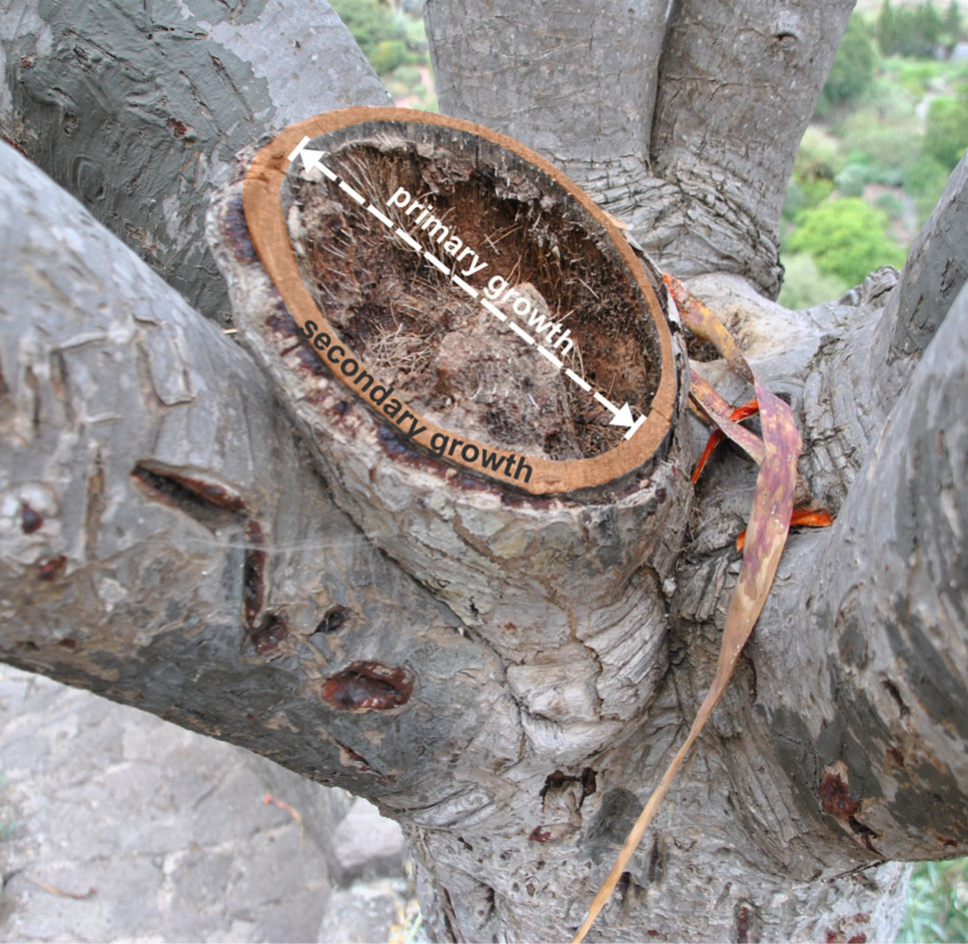
*Dracaena draco* tree with excised branch (Jardín Botánico Canario “Viera y Clavijo”, Gran Canaria, Spain). The central part of the scar is of primary origin and has collapsed, while that which is of secondary origin forms a rigid cylinder

In *Dracaena* spp., the main function of secondary tissue is that of mechanical support, since, in its absence, the primary body would be unstable (Tomlinson 1964). As can be seen in Fig. 4, a narrow and rigid peripheral cylinder formed by secondary growth supports a thick branch of the *D. draco* tree. Stem tracheids of this species possess characteristics typical of fibres. Unlike conifer tracheids, which usually do not elongate (Bailey 1920; Lewis 1935), these tracheids are very long due to intrusive elongation. As pointed out by Carlquist (1975, 2001), the greater length of tracheary elements provides greater strength. Moreover, the arrangement of tracheids in *D. draco* stem contributes to mechanical stability, as these cells do not form a straight column within the vascular bundle, rather, they are strongly displaced from each other, or even interwoven thus forming a braid-like arrangement. The presence of irregularly arranged protrusions along the tracheid body possibly stabilizes the entire structure by occupying intercellular spaces following bundle formation. Additionally, as mentioned above, the bundles may undergo uniting during development, thereby contributing to the formation of a more complex and rigid network of tracheids.

Xylem configuration also provides physiological information. Carlquist (2012) indicated that the wide diameter of monocot tracheids may compensate for the fact that vessels are absent from secondary bundles. In turn, the presence of tracheids (conductive imperforate tracheary elements) can be suggested to be more cavitation resistant than vessels (Sano et al. 2011). According to Waterhouse (1987), pitting of the tracheary elements of dracaenoid plants (*Cordyline stricta*, *Aloe* sp., *Yucca* sp. and *Xanthorrhoea australis*) is rather like the condition usually found in fibres. In true fibres, however, the pits are restricted to the median part of fibres (Esau 1965), while in the investigated *D. draco*, as well as other species studied by Waterhouse (1987), pits also occur in end walls. It is also important to emphasize that the tracheids of *D. draco* stem do not join end-to-end. Contact areas here involve the end of one tracheid overlapping the body of another. Such distribution of pits may also have mechanical significance, since abundant bordered pits in all tracheid walls would weaken the cell and compromise its mechanical function (Kedrov 2012).

To conclude, in the arborescent monocot *D. draco,* tracheids present in the secondary bundles have features in common with fibres. Their considerable intrusive growth and formation of protrusions along the tracheid body, resulting in a braid-like arrangement of tracheids within vascular bundles, together with uniting and separation of bundles, led to the formation of a complex and rigid network. The complexity of this network of tracheids, that functions both in transport and mechanical support, seems to have a major impact on the tree-like growth form of *D. draco*.

#### *Author contribution statement*

All research and the writing was done by the author.
